# Intravascular large B-cell lymphoma presenting as unilateral lymphedema in a patient with rheumatoid arthritis and sarcoidosis: A case report

**DOI:** 10.1097/MD.0000000000051323

**Published:** 2026-10-30

**Authors:** Masatoshi Kimura, Haruka Sasaki, Risa Kudo, Masayuki Murai, Yuki Rikitake, Chihiro Iwao, Kousho Iwao, Ayako Aizawa, Takeshi Kawaguchi, Motohiro Matsuda, Shunichi Miyauchi, Ichiro Takajo, Takumi Kiwaki, Kunihiko Umekita, Taiga Miyazaki

**Affiliations:** a Division of Respirology, Rheumatology, Infectious Diseases, and Neurology, Department of Internal Medicine, Faculty of Medicine, University of Miyazaki, Miyazaki, Japan; b Section of Oncopathology and Morphological Pathology, Department of Pathology, Faculty of Medicine, University of Miyazaki, Miyazaki, Japan.

**Keywords:** intravascular large B-cell lymphoma, lymphedema, random skin biopsy, rheumatoid arthritis, sarcoidosis

## Abstract

**Rationale:**

Intravascular large B-cell lymphoma (IVLBCL) is an uncommon subtype of diffuse large B-cell lymphoma, typically presenting with nonspecific symptoms and lacking lymphadenopathy or visible tumor lesions, which makes early diagnosis challenging.

**Patient concerns:**

A 66-year-old female developed progressive unilateral lymphedema despite ongoing treatment for rheumatoid arthritis and sarcoidosis. Sarcoidosis-related lymph node lesions were also considered as a potential cause, and the prednisolone dose was increased. Despite these measures, the edema worsened, and a fever of around 38°C developed. At the initial evaluation, the serum soluble interleukin-2 receptor level was already elevated at 9985 U/mL.

**Diagnoses:**

Initial investigations, including imaging, bone marrow aspiration, and random skin biopsies, failed to reveal malignancy. Subsequent monitoring revealed that the serum level of serum soluble interleukin-2 receptor had risen further to 19,835 U/mL and that thrombocytopenia was present, prompting a repeat random skin biopsy that ultimately confirmed IVLBCL through the identification of intravascular cluster of differentiation 20-positive lymphoma cells.

**Interventions:**

Chemotherapy with rituximab, cyclophosphamide, doxorubicin, vincristine, and prednisone was initiated. However, the patient developed peripheral neuropathy, suspected to be an adverse drug reaction. Consequently, the regimen was switched to polatuzumab vedotin, rituximab, cyclophosphamide, doxorubicin, and prednisolone.

**Outcomes:**

After completing 8 courses, the patient achieved remission. The left lower-limb lymphedema improved significantly.

**Lessons:**

This case highlights several clinically significant points: first, IVLBCL can present solely as unilateral lymphedema without detectable lymph node enlargement or tumorous lesions, underscoring the diagnostic difficulty. Second, repeated random skin biopsies, including adipose-rich vascular tissue, are crucial in establishing a definitive diagnosis when initial biopsies are inconclusive. Third, patients with underlying autoimmune conditions such as rheumatoid arthritis and sarcoidosis, particularly those receiving immunosuppressive therapies, may have an increased susceptibility to lymphoma, suggesting that systemic immune dysregulation could contribute to disease onset.

## 1. Introduction

Intravascular large B-cell lymphoma (IVLBCL) is a rare subtype of diffuse large B-cell lymphoma that primarily affects middle-aged and elderly individuals. It is characterized by the proliferation of neoplastic lymphoid cells within the lumina of small- to medium-sized blood vessels.^[1]^ The disease presents with a wide spectrum of nonspecific clinical symptoms, including fever, night sweats, and signs of multi-organ dysfunction. Because it typically lacks lymphadenopathy or overt tumor formation, early diagnosis is challenging, and many cases are identified only postmortem.

Lymphedema, defined as the accumulation of interstitial fluid resulting from impaired lymphatic drainage, is commonly secondary to malignancies.^[2,3]^ However, malignant lymphomas, including IVLBCL, account for <0.5% of secondary lymphedema cases,^[4]^ making this association exceedingly rare.

Here, we report a case of unilateral lymphedema that developed during treatment for rheumatoid arthritis and sarcoidosis, which was ultimately diagnosed as IVLBCL.

## 2. Case presentation

A 66-year-old female had been diagnosed with sarcoidosis 24 years earlier after presenting with uveitis and hilar lymphadenopathy. Seventeen years ago, she developed complete atrioventricular block and received a pacemaker for cardiac sarcoidosis. Around the same time, she was also diagnosed with chronic kidney disease. Two years before presentation, she developed shoulder joint pain and was diagnosed with rheumatoid arthritis based on positive anticyclic citrullinated peptide antibodies. Three months before presentation, treatment with abatacept (ABT) and prednisolone (PSL) 5 mg/d was initiated and maintained, achieving remission of rheumatoid arthritis and stable control of sarcoidosis without further organ involvement.

However, marked pitting edema developed, beginning in the left thigh and extending distally. Suspecting drug-induced edema, ABT was discontinued. Sarcoidosis-related lymph node lesions were also considered as a potential cause, and the PSL dose was increased to 10 mg/d. Despite these measures, the edema worsened, and a fever of around 38°C developed.

Laboratory investigations revealed neutrophil-predominant leukocytosis and normocytic anemia. C-reactive protein levels were within normal limits, and angiotensin-converting enzyme levels were not elevated. However, serum soluble interleukin-2 receptor (sIL-2R) was markedly elevated at 9985 U/mL (Table 1). A noncontrast computed tomography (CT) scan of the lower extremities showed no tumorous lesions or lymph node enlargement that could explain vascular compression. Lymphoscintigraphy demonstrated impaired lymphatic flow from the left lower leg to the thigh and dermal backflow predominantly along the medial aspect of the left lower leg and thigh (Fig. 1). These findings confirmed the presence of lymphedema, but the underlying cause of lymphatic obstruction remained unclear.

**Table 1:** Laboratory findings at initial presentation.

Blood count	Patient value	Normal range	Units	Biochemistry	Patient value	Normal range	Units
WBC	10.9	3.3–8.6	×10^3^/μL	TP	5.91	6.6–8.1	g/dL
Neut.	92.6	37–72	%	Alb	3.21	4.1–5.1	g/dL
Lymph.	3.7	20–50	%	BUN	63.3	8–20	mg/dL
Mono.	3.4	4.1–10.6	%	Cre	4.46	0.46–0.79	mg/dL
Eosino.	0.2	0.6–8.3	%	T-Bil	0.5	0.4–1.5	mg/dL
Baso.	0.1	0–1.3	%	AST	14	13–30	U/L
RBC	277	386–492	×10^4^/μL	ALT	12	7–23	U/L
Hb	8.6	11.6–14.8	g/dL	LDH	277	124–222	U/L
Ht	26.8	35.1–44.4	%	γ-GTP	15	9–32	U/L
Plt	22	15.8–34.8	×10^4^/μL	ALP	89	38–113	U/L
				CK	37	41–153	U/L
ESR	84	3–15	mm/h	CRP	0.07	0–0.14	mg/dL
				BNP	25.1	<18.4	pg/mL
Coagulation				ACE	9.7	7–25	U/L
PT	12	11.4–14	s	Lysozyme	18.8	5–10.2	μg/mL
PT%	121.5	80–100	%	sIL-2R	9985	204–587	U/mL
PT-INR	0.89	0.8–1.2					
APTT	25.2	22.5–37.5	s				
D-dimer	1.33	<1	μg/mL				

ACE = angiotensin-converting enzyme, Alb = albumin, ALP = alkaline phosphatase, ALT = alanine aminotransferase, APTT = activated partial thromboplastin time, AST = aspartate aminotransferase, Baso. = basophil, BNP = brain natriuretic peptide, BUN = blood urea nitrogen, CK = creatine kinase, Cre = creatinine, CRP = C-reactive protein, Eosino. = eosinophil, ESR = erythrocyte sedimentation rate, Hb = hemoglobin, Ht = hematocrit, LDH = lactate dehydrogenase, Lymph. = lymphocyte, Mono. = monocyte, Neut. = neutrophil, Plt = platelet, PT = prothrombin time, RBC = red blood cell, sIL-2R = soluble interleukin-2 receptor, T-Bil = total bilirubin, TP = total protein, WBC = white blood cell, γ-GTP = gamma-glutamyl transpeptidase.

**Figure 1.**
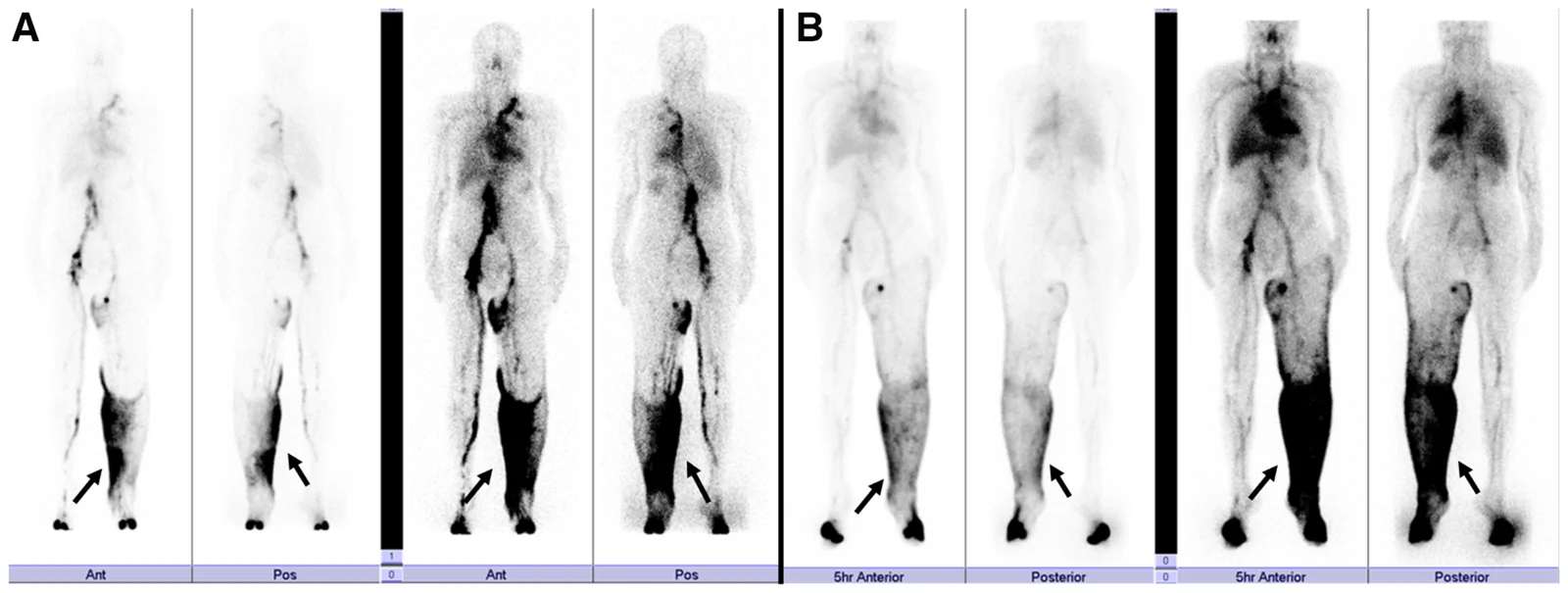
Lymphoscintigraphy. (A) Image at 1 hour and (B) image at 5 hours. Lymphatic flow from the left lower leg to the thigh is impaired. Dermal backflow is prominent along the medial aspects of the left lower leg and thigh.

The elevated sIL-2R levels raised suspicion for malignant lymphoma, including IVLBCL. Bone marrow aspiration and random skin biopsies from clinically normal skin on the abdomen and both thighs revealed no histological evidence of malignancy. Positron emission tomography-CT also showed no abnormal fluorodeoxyglucose uptake in lymph nodes or other organs. Because these investigations revealed no findings suggestive of malignancy, we considered the possibility of drug-induced or sarcoidosis-related lymphedema. The patient continued supportive care with elastic compression bandages and manual lymphatic drainage during rehabilitation. In addition, because purulent urine was observed, a urinary tract infection was suspected as a possible cause of the fever, and antibiotics were administered. During treatment, clinical symptoms and laboratory findings of the patient, including sIL-2R levels, were carefully monitored. Although the unexplained fever persisted, the lower extremity edema did not worsen.

Five months later, serum sIL-2R levels further increased to 19,835 U/mL, and thrombocytopenia was observed (Fig. 2). Based on these changes in objective findings, we reconsidered the possibility of a hematologic malignancy, including malignant lymphoma. A second random skin biopsy was performed, which revealed clusters of cluster of differentiation (CD)20-positive large lymphocyte-like cells within the blood vessels of subcutaneous adipose tissue obtained from the right lower abdomen (Fig. 3). These findings established the diagnosis of IVLBCL. In addition to CD20, CD10, CD79a, B-cell lymphoma 6, and MUM1 were also evaluated. Because the results were CD10(−), CD79a(+), B-cell lymphoma 6(+), and MUM1(+), respectively, the phenotype was consistent with a nongerminal center B-cell. Although follow-up plain CT and positron emission tomography-CT scans were performed after the diagnosis, no obvious lesions that compressed lymphatic vessels or veins were found, including in the left pelvis and retroperitoneal region.

**Figure 2.**
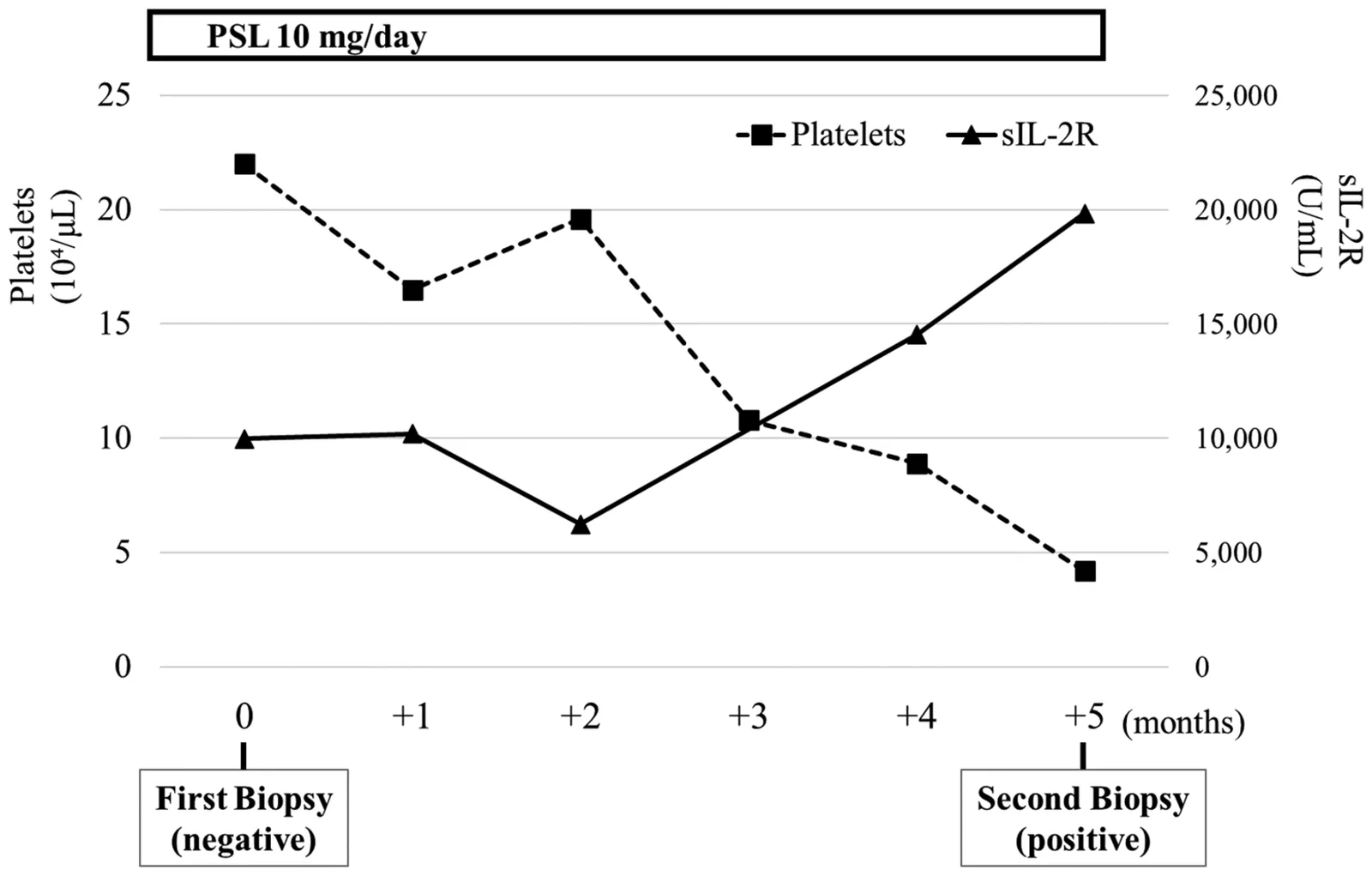
Clinical course of platelet counts and sIL-2R levels. The first random skin biopsy was negative for IVLBCL, whereas the second biopsy, performed after a further increase in sIL-2R levels and the development of thrombocytopenia, was positive, leading to the diagnosis of IVLBCL. IVLBCL = intravascular large B-cell lymphoma, PSL = prednisolone, sIL-2R = soluble interleukin-2 receptor.

**Figure 3.**
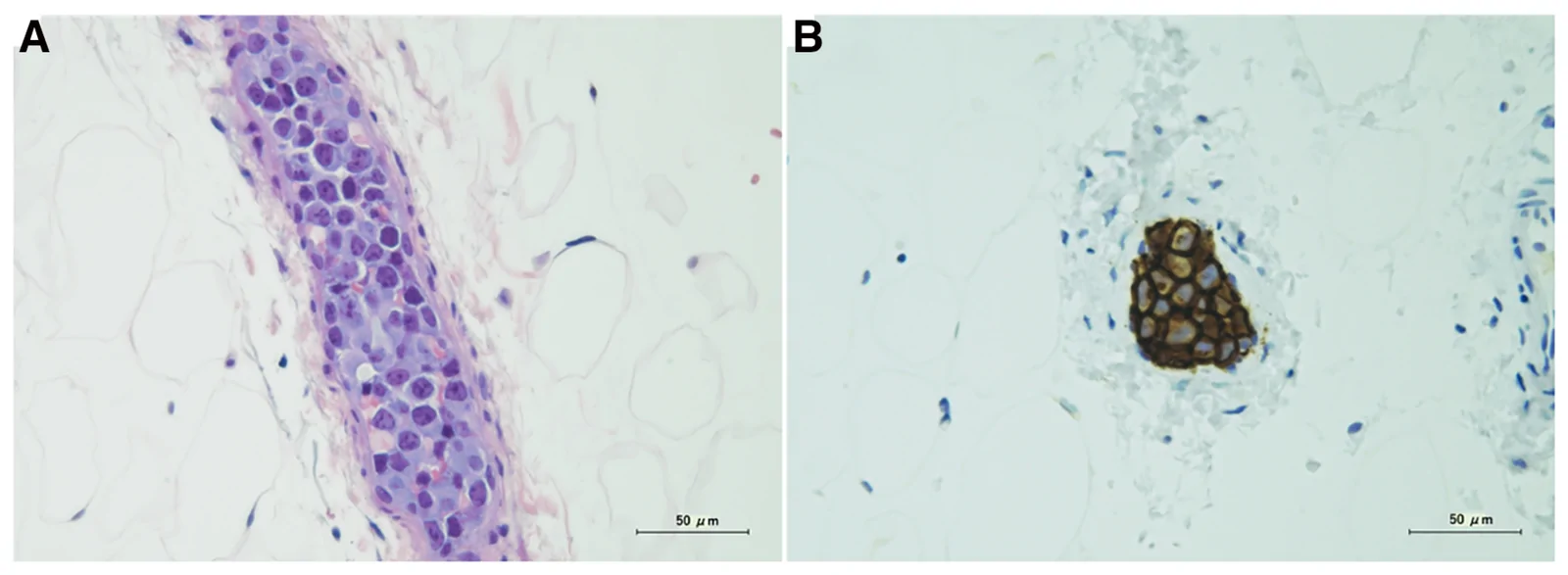
Random skin biopsy pathology. (A) Hematoxylin and eosin staining, 40× magnification. Clusters of large lymphocyte-like cells are observed within blood vessels. (B) CD20 immunohistochemical staining, 40× magnification. The large lymphocyte-like cells are CD20-positive.

Chemotherapy with rituximab, cyclophosphamide, doxorubicin, vincristine, and prednisone was initiated. Despite the preexisting renal impairment, no renal function-based dose reductions of the antineoplastic agents were required, and standard doses were administered. In addition, to prevent tumor lysis syndrome and further deterioration of renal function, we administered chemotherapy while continuing fluid replacement at a rate of 1 L per day. However, after the first cycle, the patient developed Common Terminology Criteria for Adverse Events Grade 3-equivalent peripheral neuropathy, suspected to be an adverse drug reaction. Because continued administration of vincristine was considered difficult, the regimen was switched to polatuzumab vedotin, rituximab, cyclophosphamide, doxorubicin, and prednisolone from the second cycle to maintain therapeutic efficacy while avoiding further exposure to vincristine. We did not reduce the dose of polatuzumab vedotin, nor did we administer supportive care such as specific drug therapy for peripheral neuropathy. Instead, we carefully monitored the symptoms of peripheral neuropathy and extended the dosing interval as needed. Following the change in treatment, the peripheral neuropathy gradually improved, and the patient completed 8 cycles of the polatuzumab vedotin, rituximab, cyclophosphamide, doxorubicin, and prednisolone chemotherapy regimen without the development of new neuropathy or worsening of existing neuropathy. The patient subsequently achieved remission. Although no further deterioration in renal function was observed during chemotherapy, no clear improvement in renal function was observed even after remission of IVLBCL, and renal impairment remained. In contrast, the left lower-limb lymphedema improved significantly following the initiation of chemotherapy, along with continued compression therapy using elastic bandages (Fig. 4).

**Figure 4.**
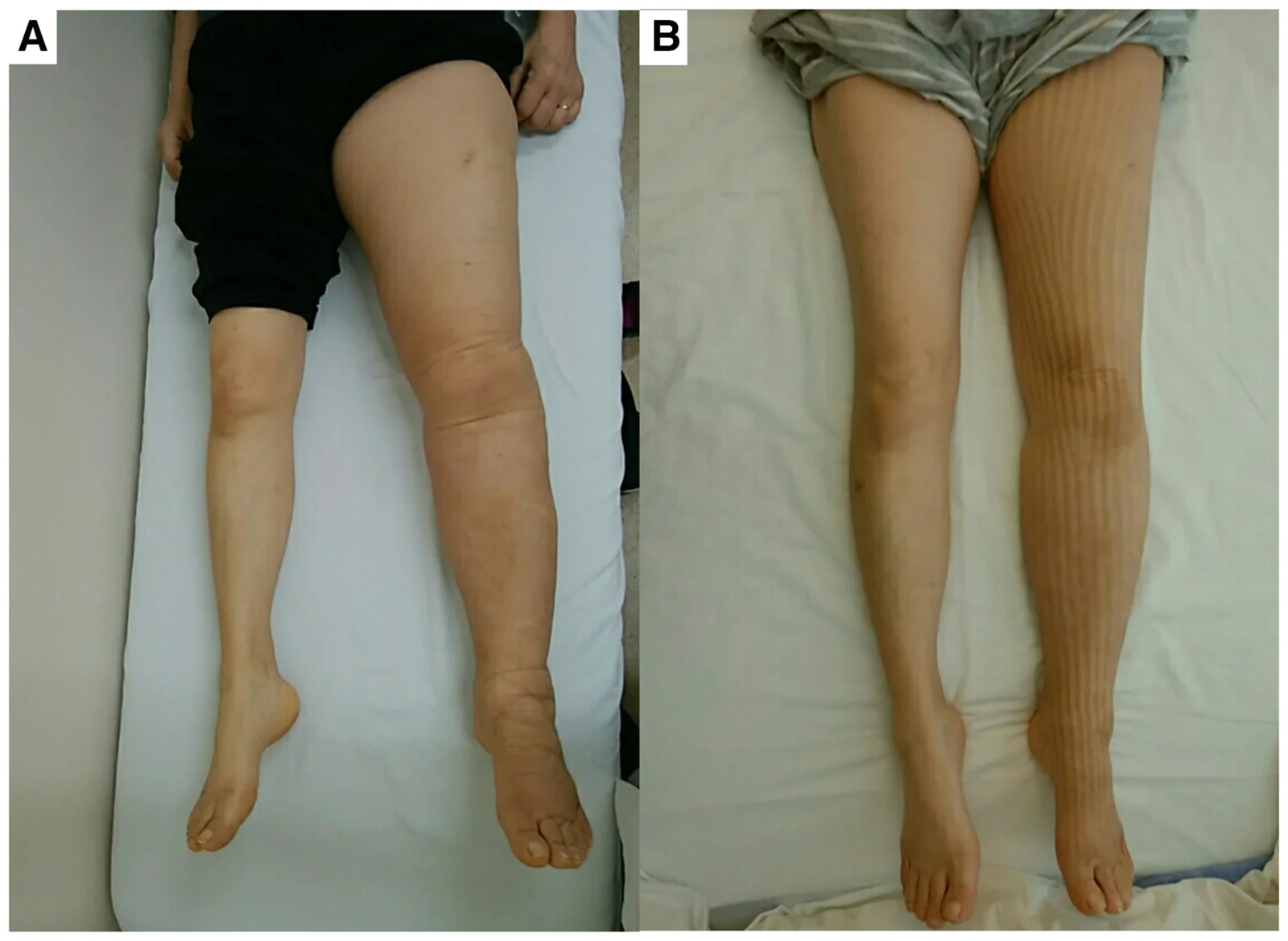
Progression of lymphedema. (A) Before treatment and (B) 2 months after initiation of therapy. Lymphedema of the left lower limb improved following Pola-R-CHP chemotherapy. Pola-R-CHP = polatuzumab vedotin, rituximab, cyclophosphamide, doxorubicin, and prednisolone.

## 3. Discussion

We encountered a patient who developed unilateral lymphedema during treatment for rheumatoid arthritis and sarcoidosis, ultimately diagnosed with IVLBCL as the underlying cause of lymphatic drainage impairment.

IVLBCL is characterized by a lack of specific clinical manifestations, making diagnosis particularly challenging. Histopathological examination is essential for confirmation, and diagnosis can sometimes be achieved through biopsy of macroscopically normal skin. In cases without visible skin lesions, random skin biopsies that include vascular structures within adipose-rich tissue are considered especially useful for detecting intravascular lymphoma cells.^[5]^ However, some studies report a diagnostic sensitivity of only about 50%.^[6]^ In this case, tumor cells were not detected in the initial skin biopsy, and the diagnosis was established only after a repeat biopsy. Both the initial and second biopsies were performed at a sufficient depth to include subcutaneous adipose tissue, with no apparent technical differences in the sampling method or specimen depth. Although the possibility that the initial biopsy failed to capture blood vessels containing tumor cells cannot be excluded, sIL-2R levels increased further and thrombocytopenia developed during the 5-month period between the initial and second biopsies. Therefore, disease progression during this period possibly resulted in an increased number of tumor cells within the blood vessels, facilitating their detection in the second biopsy. As demonstrated in this case, a negative initial random skin biopsy does not completely rule out IVLBCL. Therefore, repeat random skin biopsy should be considered if clinical suspicion persists or if new findings suggestive of IVLBCL emerge during follow-up. Furthermore, although the lymphedema was confined to the left lower limb, the tumor cells that led to the diagnosis of IVLBCL were detected in a skin biopsy specimen obtained from the right lower abdomen, where no clinical findings, such as edema or rash, were present. This finding highlights the limitations of selecting biopsy sites solely on the basis of localized clinical symptoms and may reflect the systemic intravascular involvement characteristic of IVLBCL. Taken together, these findings further support the diagnostic value of random skin biopsy of clinically normal-appearing skin.

Unilateral lower-limb lymphedema is a rare initial presentation of malignant lymphoma. A previous report described a case in which inguinal lymph node swelling compressed the iliac vein, causing unilateral lower-limb lymphedema.^[7]^ In this case, because imaging findings revealed no lymph node enlargement or obvious lesions that compressed lymphatic vessels or veins, the relationship between IVLBCL and lymphedema was initially unclear. However, the marked improvement of edema following chemotherapy strongly suggested that IVLBCL was responsible for the lymphatic obstruction. Pallure et al^[8]^ reported a case of IVLBCL presenting with bilateral lower-limb lymphedema and fever, diagnosed through a skin biopsy from the lower limb. Although our case differed from previous reports in that the lymphedema was unilateral and malignant cells were detected in biopsy specimens from the abdomen, lymphoscintigraphy revealed an obstruction of lymph flow from the left lower leg to the thigh. Therefore, it was suggested that localized microcirculatory or lymphatic dysfunction associated with IVLBCL may have contributed to the unilateral lymphedema. However, it is difficult to identify the exact mechanism based solely on the findings from this case.

This patient also had rheumatoid arthritis and sarcoidosis, both of which are rheumatic diseases that can occasionally cause lymphedema. In rheumatoid arthritis, lymphedema is thought to result from fibrosis of superficial lymphatic vessels secondary to chronic synovitis, leading to impaired drainage.^[9]^ Improvement has been reported with glucocorticoid or biologic therapy.^[10]^ In sarcoidosis, lymphatic edema may occur due to lymph node swelling or tenosynovitis, and glucocorticoids are generally effective in such cases.^[11]^ In our patient, however, lymphedema developed during biologic therapy and did not improve despite increasing the PSL dose, suggesting that rheumatoid arthritis and sarcoidosis were unlikely causes.

Patients with rheumatoid arthritis have an approximately 2-fold higher risk of developing non-Hodgkin lymphoma compared with the general population,^[12]^ and this risk has been shown to correlate with rheumatoid arthritis disease severity.^[13]^ In addition, methotrexate and other immunosuppressive agents used in rheumatoid arthritis management have been associated with lymphoproliferative disorders, including malignant lymphoma.^[14]^ Furthermore, Brincker proposed the concept of sarcoidosis–lymphoma syndrome in 1986, noting that patients with sarcoidosis have a 5.5-fold higher incidence of lymphoma than the general population.^[15]^ A case of IVLBCL occurring during sarcoidosis treatment with generalized lymphadenopathy and cardiac involvement has also been reported.^[16]^ In the present case, the patient had received the biologic agent ABT for rheumatoid arthritis and had multi-organ sarcoid involvement, including the eyes, lungs, and heart. Some reports have shown that the use of ABT did not significantly increase the risk of malignant tumors, including malignant lymphoma, compared to other drugs or the general population.^[17,18]^ Although the effect of ABT-mediated immune modulation on tumor immunosurveillance cannot be completely excluded, a causal relationship between ABT and IVLBCL cannot be inferred from this single case. Therefore, it is possible that systemic immune dysregulation associated with the underlying rheumatic diseases and their treatment contributed to the onset of IVLBCL.

This case illustrates that IVLBCL can present as unilateral lymphedema without apparent lymph node enlargement or detectable lesions on imaging, making diagnosis particularly difficult. Repeated random skin biopsies were essential in achieving the correct diagnosis, and prompt initiation of chemotherapy led to remission. In addition, the potential contribution of rheumatoid arthritis, sarcoidosis, and their associated immune abnormalities to the development of IVLBCL cannot be excluded. When unexplained lymphedema arises during treatment for rheumatoid arthritis or sarcoidosis, clinicians should consider IVLBCL in the differential diagnosis and perform repeated diagnostic evaluations, including random skin biopsies, even in the absence of overt lymphadenopathy.

## Acknowledgments

We acknowledge Enago (https://www.enago.jp/) for English language editing.

## Author contributions

**Data curation:** Masatoshi Kimura, Haruka Sasaki, Risa Kudo, Masayuki Murai, Yuki Rikitake, Chihiro Iwao, Kousho Iwao, Ayako Aizawa, Takeshi Kawaguchi, Motohiro Matsuda, Shunichi Miyauchi, Ichiro Takajo.

**Formal analysis:** Takumi Kiwaki.

**Supervision:** Kunihiko Umekita, Taiga Miyazaki.

**Writing – original draft:** Masatoshi Kimura, Haruka Sasaki.

**Writing – review & editing:** Kunihiko Umekita, Taiga Miyazaki.
